# Special Issue “Advanced Spectroscopy Techniques in Food Analysis: Qualitative and Quantitative Chemometric Approaches”

**DOI:** 10.3390/foods12152831

**Published:** 2023-07-26

**Authors:** Mourad Kharbach, Samuli Urpelainen

**Affiliations:** 1 Department of Food and Nutrition, University of Helsinki, 00014 Helsinki, Finland; 2 Department of Computer Sciences, University of Helsinki, 00560 Helsinki, Finland; 3 Nano and Molecular Systems Research Unit, University of Oulu, 90014 Oulu, Finland

The globalization of the food market has created a pressing need for food producers to meet the ever-increasing demands of consumers while ensuring adherence to stringent food safety and quality standards [1]. The comprehensive analysis of food quality encompasses numerous aspects, such as chemical characterization, physical properties, sensory evaluation, authentication, traceability, processing, storage, and microbiological safety [2]. Traditional analytical techniques have long been employed in food analysis, but they often involve destructive procedures that are labor-intensive, time-consuming, costly, and environmentally burdensome [3].

In response to these challenges, the field of food analysis has witnessed remarkable advancements through the utilization of advanced spectroscopic techniques. These cutting-edge methods, including X-ray-based approaches, hyperspectral and multispectral imaging, NMR, Raman, IR, mass, UV, visible, and fluorescence spectroscopy, offer non-destructive, rapid, solvent-efficient, eco-friendly, and cost-effective alternatives to conventional methods [4]. Leveraging these techniques in tandem with statistical analysis, particularly through chemometric approaches, allows for the extraction and exploration of vital information hidden within spectral fingerprints or image data. Furthermore, this extracted information can be utilized to construct calibration models for qualitative and quantitative analysis of various food samples. The integration of advanced spectroscopy and chemometrics holds immense potential in the field of food science and technology, bolstering consumer confidence and contributing to overall food quality assurance [3].

It is with great pleasure that we present this Special Issue, which focuses on recent developments and applications of advanced spectroscopic techniques in food analysis, quality evaluation, safety assessment, and practical industrial implementations, with a specific emphasis on chemometric approaches. The collection of papers included in this Issue offers a valuable insight into the diverse range of research and applications in this field, shedding light on the potential of these techniques to revolutionize food analysis.

The accepted papers cover a broad spectrum of topics within the scope of this Special Issue. The first paper presents a comprehensive review of the current applications of advancing spectroscopy techniques in food analysis, focusing on the data handling aspect with chemometric approaches [3]. This review offers an overview of the progress made in the field and identifies avenues for further research and development.

Furthermore, one paper details an innovative application of laser-induced breakdown spectroscopy coupled with variable selection algorithms and chemometrics for the detection of heavy metals in *Fritillaria thunbergia* [5]. Another paper delves into the phenotypic analysis of Fourier-transform infrared milk spectra in dairy goats, providing valuable insights into the characterization and quality assessment of dairy products [6]. Additionally, the utilization of spatial frequency domain imaging and machine learning for the rapid and accurate detection of bruised tissue in pears is explored, highlighting the potential of these techniques for quality control purposes [7].

In another study, the discrimination of Brazilian stingless bee honey based on its iron-based biogeographical origin is investigated, showcasing the applicability of discriminant analysis in ensuring the authenticity and traceability of food products [8]. The quality evaluation of fair-trade cocoa beans from different origins using portable near-infrared spectroscopy (NIRS) is also examined, illustrating the potential of NIRS as a non-destructive tool for rapid quality assessment in the cocoa industry [9].

Additionally, the effect of moisture content on the analysis of quality attributes of red pepper powder is explored using a hyperspectral system, providing valuable insights into the impact of moisture on food analysis outcomes [10]. Moreover, time-resolved laser-induced breakdown spectroscopy is employed for the accurate qualitative and quantitative analysis of brown rice flour adulteration, offering a promising approach to combat food fraud and adulteration [11].

Furthermore, the classification of *Prunus* genus by botanical origin and harvest year based on carbohydrates profiles is investigated, shedding light on the application of spectroscopic techniques for the authentication of botanical products [12]. The chemical authentication and speciation of *Salvia* botanicals are explored using GC/Q-ToF and chemometrics, providing crucial insights into the identification and characterization of herbal products [13].

Lastly, the detection of pesticide residue levels in grapes is studied using hyperspectral imaging and machine learning, illustrating the potential of these techniques for ensuring food safety [14].

In conclusion, this Special Issue brings together a collection of research papers that highlight the immense potential of advanced spectroscopic techniques in the field of food analysis and quality evaluation. By presenting a range of innovative applications, these studies demonstrate the power of these techniques to enhance food safety, authenticity, and overall quality. We hope that the papers in this Special Issue provide valuable insights, inspire further research, and encourage the adoption of advanced spectroscopic techniques in the food industry.
